# Polygenic risk scores in pharmacogenomics: opportunities and challenges—a mini review

**DOI:** 10.3389/fgene.2023.1217049

**Published:** 2023-06-15

**Authors:** Aurélien Simona, Wenyu Song, David W. Bates, Caroline Flora Samer

**Affiliations:** ^1^ Division of Clinical Pharmacology and Toxicology, Geneva University Hospitals and Faculty of Medicine, Geneva, Switzerland; ^2^ Division of General Internal Medicine, Department of Medicine, Brigham and Women’s Hospital, Harvard Medical School, Boston, MA, United States; ^3^ Department of Health Policy and Management, Harvard T. H. Chan School of Public Health, Boston, MA, United States

**Keywords:** polygenic risk score (PRS), pharmacogenomics, functional genomic, precision medicine, clinical decision support (CDS), adverse drug reaction (ADR)

## Abstract

Pharmacogenomics (PGx) aims at tailoring drug therapy by considering patient genetic makeup. While drug dosage guidelines have been extensively based on single gene mutations (single nucleotide polymorphisms) over the last decade, polygenic risk scores (PRS) have emerged in the past years as a promising tool to account for the complex interplay and polygenic nature of patients’ genetic predisposition affecting drug response. Even though PRS research has demonstrated convincing evidence in disease risk prediction, the clinical utility and its implementation in daily care has yet to be demonstrated, and pharmacogenomics is no exception; usual endpoints include drug efficacy or toxicity. Here, we review the general pipeline in PRS calculation, and we discuss some of the remaining barriers and challenges that must be undertaken to bring PRS research in PGx closer to patient care. Besides the need in following reporting guidelines and larger PGx patient cohorts, PRS integration will require close collaboration between bioinformatician, treating physicians and genetic consultants to ensure a transparent, generalizable, and trustful implementation of PRS results in real-world medical decisions.

## Introduction

Precision medicine aims at tailoring disease prevention and management to identify optimal therapies by considering individual factors, such as genetic profile, health conditions, environmental exposure, and lifestyle. Although very promising, developing and reaping the rewards of precision medicine has proven to be a challenging task, as it requires dealing with the complex interplay between many non-linear associations from factors that result in individual variability.

Many studies have shown that many complex diseases, such as mental disorders, addiction, pain responses, etc., can have strong genetic underpinnings. By considering patient-level genetic information, such as significant genomic loci and associated genes, genomic medicine is a rapidly emerging field that is dedicated to improve healthcare practice using an individual’s genomic information (NHGRI website, 2021). Over the last decade, numerous genome-wide association studies (GWAS) have explored the contribution of inherited variants to common complex disorders involving many genes and their variants. While many monogenic diseases have been discovered and some have been successfully managed, diseases with complex inheritance—the majority of diseases in terms of population burden—are more difficult to tackle. Both the influence of environmental factors in disease expression and the interplay between polygenic predispositions limit the ability to predict phenotypes (disease, drug-related endpoints, etc.) probability based on genome analysis alone.

An adverse drug reaction (ADR) can be defined as “an appreciably harmful or unpleasant reaction resulting from an intervention related to the use of a medicinal product; adverse effects usually predict hazard from future administration and warrant prevention, or specific treatment, or alteration of the dosage regimen, or withdrawal of the product” (Aronson and Ferner, 2005). In addition to poor or even non-response to treatment, ADRs cause a significant burden in healthcare and where estimated in 1998 to represent the fourth leading cause of death in the United States (Lazarou et al., 1998). Moreover, differences in drug response are partially determined by genetic factors (Pirmohamed, 2014; García-González et al., 2016), as illustrated by different dosage regimen according to specific polymorphisms in metabolic enzymes like the cytochrome P450 superfamily (Caudle et al., 2017).

Pharmacogenomics (PGx) is the study of how an individual’s genetic makeup affects the response to drugs. It can contribute to the development of precision medicine by selecting drugs with better efficacy while lowering the risk of ADRs during clinicians’ prescription decision making process. Many drug-gene pairs are currently used in clinical practice to help in finding the beneficial regimen for a specific patient with a specific condition. Evidence-based guidelines have been developed for more than a decade to advise prescribers in daily clinical practice on an optimal drug regimen according to genotypes or predicted phenotypes (Swen et al., 2011; Caudle et al., 2017). Unfortunately, this conventional approach of drug response prediction based on a single PGx variant biomarker depends on large effect size and the absence of comorbidities modulating drug response. In many cases, it cannot address the polygenic nature of most observable drug response outcomes where the effects of individual genetic variants are small. Many studies have showed the limitation of individual genetic marker association models, which can only explain small amounts of phenotypic variation of patients, resulting in so-called “missing heritability” (Eichler et al., 2010).

## Polygenic risk scores

One promising approach in PGx is the use of patient-level polygenic risk scores (PRS). In simple terms, PRS reflects the genetic predisposition for a phenotype of interest. More precisely, PRS calculates the sum of genome-wide risk alleles that are weighted by their corresponding effect size estimates (e.g., odds ratio) derived from GWAS summary statistic data (Choi et al., 2020). Importantly, this additive property ignores any gene-environment or gene-gene interactions (Lewis and Vassos, 2020). PRS has a significant higher capacity to predict risks of complex diseases and other health-related conditions by considering small impacts of many variants from genome-wide scope in a single numeric index, making it efficient and convenient for large-scale population risk screening and disease diagnosis prediction.

## Polygenic risk scores in disease risk prediction

The clinical utility of PGS has already been highlighted in recent research. For example, Khera et al. (Khera et al., 2018) demonstrated that PRS used in coronary artery disease was shown to predict a risk comparable to the one caused by rare and highly penetrant monogenic mutations, but with the ability to generalize to a broader population. In addition, atrial fibrillation, type 2 diabetes, breast cancer, and inflammatory bowel disease represent other common conditions in which populations have been identified as having disease risk at least three-fold higher than the general population, with a proportion of identified individuals ranging from 1.5% to 8% depending on the disease. Even though these effects may sound modest compared to some clinical factor-based indices, they may also be more clinically relevant as a genetic tool since, compared to monogenic mutations, a higher proportion of individuals can be classified with a high risk of disease. Further, osteoporotic fractures prediction using PRS was able to decrease by 41% the proportion of patients requiring screening tests, while maintaining sensitivity and specificity high enough to identify individuals who could benefit from an intervention. This also illustrates how negative prediction could be exploited with genetically more complex diseases that lead to a rarity of events and therefore often lower precision due to a low cases/controls’ ratio. This approach is likely to prove beneficial in PGx, not only in positive prediction by ensuring optimal therapy for each patient (the right medication to the right person at the right time), but also to prevent use of ineffective drugs. Thus, using PRS to avoid unnecessary prescription should be a primary concern, both for medical and economic reasons as preventing ADR rates, improving therapeutic success, and reducing time to find optimal drug regimen is cost-effective at patient and healthcare levels.

## Polygenic risk scores in PGx

Like what has been done in genomic medicine, most progress from pharmacogenetic studies has allowed to identify rare mutations carrying high-risk of poor outcomes. Some examples of common drug-variant pairs implemented in clinical practice to decrease ADR risk are abacavir and HLA-B*5701, thiopurines (e.g., 6-mercaptopurine) and TMPT, among many others. It is also well established that allelic variations of genes involved in drug elimination, such as CYP2D6, can impact drug response both in terms of efficacy and toxicity depending on the metabolic profile of specific drugs. Moreover, predicting PGx endpoints like pain response with a polygenic polymorphisms combination and related gene set involved both in drug response (OPRM1) and disposition (ABCB1, COMT) has already been proposed (Lötsch and Geisslinger, 2006; Campa et al., 2008).

While showing great promise, the potential impact of PRS in PGx remains largely unknown. This may be not surprising since using PRS with PGx endpoints (safety or efficacy) is more challenging. High fidelity of patients’ response phenotypes to drugs is one of the major limiting factors for this type of research. In addition to compare uniformly treated patients, PGx studies must rely on well-defined endpoints (e.g., clinical scales for specific disorders like depression) which requires patient data with high level of granularity in a strictly-defined time duration. This type of information is often only available or recorded patient-level clinical databases. Another difficulty resides in polypharmacy (i.e., co-administration of multiple drugs - usually more than 5 - in a specific patient) that increases the risk of drug-drug interaction, or underlying diseases like kidney failure, both may increase susceptibility to toxicity and then modulate estimation of genetic effect sizes.

More specifically, a review by (Johnson et al., 2022) found that most published research focused on the treatment of psychiatric disorders (*n* = 31/50), followed by circulatory and digestive conditions. Among the 105 drug phenotypes that were analyzed, 82 were related to drug efficacy and response and 23 were related to ADR phenotypes. Phenotypes for which GWAS summary data were used to compute PRS were grouped in three distinct categories, i) disease-related phenotype (e.g., PRS for antipsychotic response using schizophrenia GWAS data), ii) pharmacogenomic-related phenotype (e.g., drug-induced phenotype), and iii) ADR-related phenotype (e.g., PRS for antipsychotic-induced weight gain using obesity GWAS data).

Cross et al. have also reviewed common drugs associated with PRS studies in ADR and efficacy prediction (Cross et al., 2022). Frequently prescribed medication like statins, clopidogrel, warfarin (more and more substituted by newer direct oral anticoagulants), antipsychotics, and antidepressants have been associated to some degree with genetic susceptibility to pharmacological endpoints. However, the contribution of PRS in explaining phenotype variance remains low, illustrating the fact that effort in PGx studies must continue.

## Calculation and optimization of PRS

As briefly mentioned before, PRS construction is based on the weighted sum of risk alleles across the genome. More specifically, PRS analyses need two independent datasets: i) a discovery (base) dataset, that is made of summary statistics (e.g., beta coefficients, odds ratio) obtained from GWAS and made available online, and ii) a target dataset, that is made of genotypes from individuals for whom we want to compute PRS. The PRS are then estimated for target samples from information obtained in discovery samples. Of note, although incorporating only genetic variants reaching genome-wide significance level may sound intuitive, improving PRS predictability by including many variants showing weaker associations with a phenotype has also been described (Cecile et al., 2019; Konuma and Okada, 2021). This approach could reflect the fact that original GWAS with limited sample size may not show sufficient power because the full heritability could be distributed over a very high number of common SNPs with very low effect size (Chatterjee et al., 2016). This limitation could result in potentially missed genuine trait-variant associations and justify parameter optimization in order to select an optimal threshold that could vary depending on the target trait (Choi et al., 2020).

SNP effect size estimation must be done cautiously given their uncertainty, the fact that not all of them influence phenotypes under study and independence between SNP is not guaranteed. Unadjusted values could result in poor estimation and high standard error. To mitigate this risk, different methods can be used to construct PRS (Choi et al., 2020; Zhai et al., 2022).

### Regularization techniques

These techniques, such as Lasso or Ridge regression, are commonly exploited (not only in PRS) to reduce (*shrink*) model complexity by adding a penalty factor to focus on more important variables. Differences between both techniques reside in the way parameters are tuned to create penalty. Briefly, LASSO regression can reduce variable effect to zero (decreasing the number of variables in a model), while Ridge regression tends to decrease large effects more than LASSO but keeping all variable values more than zero. While combinations of LASSO and Ridge exist, the most appropriate regression methods that should be applied may depend on the underlying phenotype under study and effect size distribution. These are very commonly in machine learning applications (not the scope of this paper), and model parameters can be optimized through iterative process.

### Clumping and thresholding (C + T) method

A potential issue can arise when summing SNP effect sizes whereas independent genetic effects do not occur, as this could be the result of non-random association of alleles at different loci correlation, a phenomenon known as linkage disequilibrium (LD) (Slatkin, 2008). This issue specifically arises when PRS is calculated from GWAS that evaluated one SNP at a time. To alleviate this correlation between polymorphisms and getting closer to independent data, SNPs can be clumped (C) by prioritizing SNP with the smallest GWAS *p*-value so that only variants that are weakly correlated are retained. Thresholding (T), the second filtering step, consists in removing variants with a *p*-value that exceeds a predetermined level of significance (Privé et al., 2019).

### Bayesian modeling

Although C + T approach looks attractive because of computational and conceptual simplicity, some authors mentioned that prediction accuracy can be limited (Vilhjálmsson et al., 2015; Ge et al., 2019). Another method that is gaining more interest relies on using prior effect size distribution to accommodate varying effect size distributions across complex traits and varying genetic architecture. Different Bayesian regression frameworks have been developed to infer the posterior mean effect size of each genetic marker from GWAS summary statistics while accounting for LD (Ge et al., 2019).

Recent simulation studies by Zhai et al. (Zhai et al., 2022) using PGx data to build PRS have compared Bayesian, C + T methods and regularization techniques. Bayesian approaches, under different genetic architectures, showed superiority to all other methods. In addition, the C + T method outperformed LASSO regression techniques, suggesting that the latter was sensitive to small signal-to-noise ratio.

## Implementation of PRS

**FIGURE 1:**
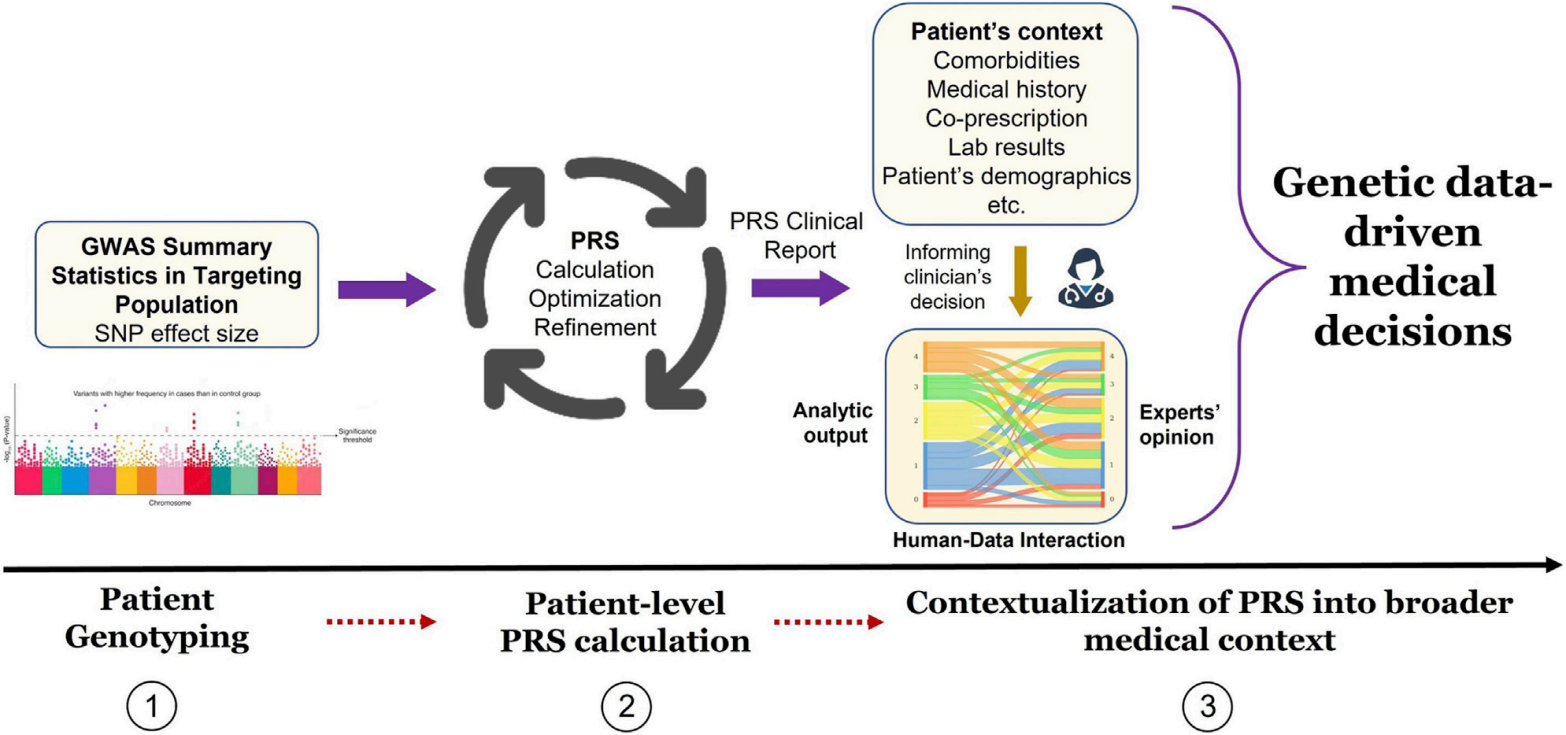
Implementation of PRS results into medical decision process. In phase 1, GWAS summary statistic data is searched for PGx endpoint (e.g., non-response to drug) effect size (e.g., odds ratio) adapted to the targeted patient genotype. In phase 2, PRS is calculated from the sum of genome-wide risk alleles that are weighted by their corresponding effect size estimates derived from GWAS data. In phase 3, PRS results are contextualized by the medical team (treating physician, genetic consultant, etc.) by incorporating patient’s medical history and all non-genetic factors (comorbidities, laboratory values, etc.) to ensure appropriate medical decisions.

Clinical implementation of PRS is a topic of active research (Shieh et al., 2017; Brockman et al., 2021). However, several steps are necessary to translate PRS promise into daily clinical routine and many hurdles still must be overcome as illustrated by the recent GenoVA study of Hao et al. who implemented PRS information in the clinic (Hao et al., 2022). In short, they proposed to conceptualize the way from PRS assay development to its clinical use as a three-step path: the first step involves epidemiology and statistical genetics and is related to PRS development, validation, and improvement; the second step involves laboratory pipeline translating PRS calculation to actionable results; the third and final step involves medical decisions where reported PRS information is integrated into patient clinical information. While PRS calculation and statistical methods are showing great progress, challenges persist in how laboratory pipeline and medical interpretation can bring PRS closer to clinical routine. An example of reporting workflow is illustrated in Figure 1.

First, a significant well-established bias resides in the predominance of European ancestry in GWAS effect size estimation, limiting the generalizability of PRS results to other populations that are already underserved (Martin et al., 2019; Tata et al., 2020; Peterson et al., 2019; Popejoy and Fullerton, 2016). Second, regardless of genetic origin, analytic validity of laboratory pipeline for clinical implementation must be ensured since the standards used for research purpose may not be sufficient. Finally, implication of both laboratory and treating physician in this process implies defining everyone’s role and area of expertise. In addition, uncertainty remains regarding the extent of information and the support that should be provided to the physician to facilitate PRS integration in medical decisions.

Converting good intentions and existing promises into concrete actions necessitates to understand clinicians’ expectations about PRS and the way to develop their trust in genetic clinical tool. As proposed by Hao et al., a transparent PRS report containing directive-free guidelines, along with evidence and limitations about PRS interpretation may be beneficial. This is important at least for two reasons: 1) in order to establish trust, laboratory choices and associated resources that were used to define risk thresholds and interpretation (e.g., dichotomous vs. continuous risk assessment) must be clearly explained, also knowing that these interpretation parameters may vary between risk phenotypes; 2) considering the current absence of demonstrated clinical utility on patient outcomes, physician support through educational resources and genetic counseling should be provided since they are responsible for making the final medical decisions. The importance of reporting informational guidelines is primordial given the need to contextualize PRS results with patient’s medical history.

## Discussion

Genetic variants are nowadays commonly analyzed in PGx, both *a posteriori* when ADR or drug unresponsiveness are suspected but also preemptively when optimal dosage of a drug with a narrow therapeutic index (i.e., ratio of the dose that produces toxicity to the dose that produces a clinically desired or effective response) must be determined as quickly as possible. However, like for many chronic conditions, pharmacological drug response depends on a myriad of factors, whether related to drug properties, patient’s pharmacokinetic profile (i.e., the way the body impact the time course of the drug in the body—metabolism playing a substantial role), or patient’s pharmacodynamic profile (i.e., the way the body responds—or not—after drugs have reached their target). We can therefore easily imagine that multiple variations in the genome can affect drug response, and this is the reason why PRS looks promising in PGx.

However, as already mentioned earlier, PGx data are not as available for PRS as is disease information. In addition, as mentioned by Kumuthini et al. (Kumuthini et al., 2022), further research is needed before widespread use in clinical practice as only near-evidence of clinical utility was identified. This is illustrated even in fast-moving fields like cancer research where evidence of clinical utility and validation of biomarkers are not always adequate (Horgan et al., 2014). Even though barriers are still to be overcome, propositions have been made to make the most of the potential of the PRS.

Even though phenotype variance explained by PRS is frequently low, this number is expected hopefully to increase with larger cohorts of patients, predominantly in ethnic population under-represented in currently available biobanks, including UK Biobank and All of US research program (Sudlow et al., 2015; All of Us Research Program Investigators Denny et al., 2019). Moreover, it has been observed that targeting the top deciles in terms of genetic risk may prove useful as this population may carry a significant higher risk (the opposite reasoning can be made for bottom deciles group). In this context, PRS may help in better stratifying patients between groups, and be a useful tool, for example, to refine screening guidelines. PRS may also gain interest in reclassifying intermediate-risk level patients defined by clinical scores into higher or lower risk group. This may lead to avoid unnecessary-yet-toxic treatment in newly low-risk patients or, on the contrary, to treat previously unidentified high-risk patients. This PRS population-centric application will contribute to amplify the role of prevention that resides at the heart of precision medicine, as well as potentially save significant costs in an already economically pressured healthcare environment. The ability to improve stratification could also be of interest in clinical trials where genetically unmatched patients after randomization were identified as a potential source of therapeutic effects confounding (Leonard et al., 2020). Adjusting for underlying differences in genetics could thus decrease trial failure and drug development costs.

An important issue has emerged due to the lack of reporting guidelines in PRS, along with gaps in data transparency and availability. As previously described, different approaches and parameters thresholds are available to estimate effect sizes. This makes difficult the comparison between PRS and some authors have proposed the Polygenic Risk Score Reporting Standards (PRS-RS) to facilitate translation of PRS research into clinical care (Wand et al., 2021; National Institute of Health, 2022).

From a clinical perspective, confidence in generalization of research-derived PRS to real-world patients would be essential to ensure appropriate medical decisions. In addition, it will be essential to determine how to deliver this information in ways that providers will find acceptable. Another issue is that careful attention must be paid in a similar way to what must be done when extrapolating the results of clinical trials to a population frequently suffering from additional comorbidities that the one used to derive GWAS effect size data. Despite the above-mentioned barriers in implementing PRS, the effort in developing PRS guidelines and promising examples of reporting workflow are preparing the ground for an effective PRS integration in electronic health records.

In conclusion, PRS have the potential to move pharmacogenomics a step further by providing a more personalized and comprehensive view of an individual’s genetic susceptibility to drug response. As illustrated by promising examples of PRS integration into clinical workflow, and while awaiting more robust genetic data across a bigger spectrum of genetic architecture, transparent information about the promise and limitations of PRS interpretation is key to help in its implementation for patient care. As PRS is expected to keep on improving in their ability to capture heritability of polygenic traits, there is good reason to expect that appropriate clinical trials will soon prove their clinical utility.
